# Tertiary cytoreduction in the setting of recurrent ovarian cancer (Review)

**DOI:** 10.3892/ol.2013.1445

**Published:** 2013-07-04

**Authors:** NASUH UTKU DOGAN, ACHIM SCHNEIDER, VITO CHIANTERA, SELEN DOGAN, POLAT DURSUN

**Affiliations:** 1 Department of Obstetrics and Gynecology, Faculty of Medicine, Akdeniz University, Antalya 07985, Turkey; 2 Department of Gynecologic Oncology, Benjamin Franklin Campus, Charité University, Berlin 12203, Germany; 3 Beyhekim State Hospital, Konya 42133, Turkey; 4 Department of Obstetrics and Gynecology, School of Medicine, Baskent University, Ankara 06570, Turkey

**Keywords:** recurrent ovarian cancer, tertiary cytoreduction, tumoral debulking

## Abstract

Ovarian cancer is the most lethal gynecological malignancy, with aggressive surgical debulking and adjuvant chemotherapy as the main treatment modalities. Optimal debulking during the primary surgery is significantly correlated with prolonged survival. As surgical techniques and chemotherapeutic agents improve, more patients with prolonged survival may face secondary and tertiary recurrences. The role of surgical debulking in secondary cytoreduction (SC) is not clearly defined and is based on retrospective series. The treatment of patients with primary or secondary recurrences generally consists of second-line chemotherapy, but may be performed on medically fit patients in certain circumstances. A limited number of studies concerning tertiary cytoreduction (TC) in cases of secondary recurrences have been published. In these studies, conventional prognostic factors for SC, including ascites, an advanced International Federation of Gynecology and Obstetrics (FIGO) stage and/or peritoneal carcinomatosis, did not apply to TC, but the post-operative residual tumor load was significant in determining the prognosis. A limited number of patients with completely-resectable tumors may have an opportunity for a maximal cytoreduction in these circumstances. TC appears to result in a favorable outcome and moderate complication rates. The surgery is an available option for patients with recurrence, in whom a complete tumor resection may be achieved.

## 1. Introduction

Ovarian cancer is the most lethal gynecological malignancy that presents at an advanced stage in 75% of patients. Aggressive surgical debulking and platinum-based adjuvant chemotherapy are the current standard treatment modalities that lead to a complete remission in the majority of patients. However, recurrences are frequently observed in up to 70% of cases (1). As the surgical techniques and systemic chemotherapeutic agents improve, more patients with prolonged survival may face secondary and tertiary recurrences. The management of these recurrences is not as well-established as it is for the primary disease. Generally, the treatment is tailored to each patient depending on the location of the recurrence, the performance of the patient, the disease-free interval, the previous response to platinum-based agents and the preference of the surgeon. The treatment usually consists of second-line chemotherapy, but surgery may be performed on medically fit patients in certain circumstances. However, the role of cytoreduction in primary or secondary recurrences remains controversial. Primary cytoreduction is a radical surgical procedure in which the aim is to reduce the tumor load to a non-visible status. Upper abdominal surgeries, including diaphragm stripping, liver resection, splenectomy and distal pancreatectomy, are classical procedures that are performed to achieve the goal of a non-visible tumor (2–4). Surgery following a primary intervention requires highly-skilled surgeons, a multidisciplinary approach and tertiary patient care facilities.

When a recurrence is detected during the follow-up period, a second surgery, termed secondary cytoreduction (SC) may be performed in a medically fit and selective patient population in certain circumstances. There are no strict criteria for selecting candidates for the surgery following primary or secondary recurrences. In the present review, the role of tertiary cytoreduction (TC) in secondary ovarian cancer recurrences will be discussed on the basis of the current literature.

## 2. Materials and methods

The present review aimed to present the current data on TC. The publications and data with regard to cytoreduction were identified using Pubmed, and relevant articles written in English were selected. There are abundant studies on SC in ovarian cancer. Using the search terms ‘ovarian cancer’ and ‘secondary cytoreduction’, 105 articles were identified that were published between 1989 and 2012. The search terms ‘ovarian cancer’ and ‘tertiary cytoreduction’ yielded 21 articles published between 1983 and 2013, of which eight papers, which were directly associated with TC, were eligible and included in the present review.

## 3. Cytoreduction in ovarian cancer

Optimal debulking in primary surgery is significantly associated with a prolonged survival. If the size of the tumor is small and/or the growth rate is fast, the tumor cells are more vulnerable to chemotherapy or radiotherapy. Reducing the tumor volume prior to chemotherapy synchronizes cellular growth and increases the bioavailable concentration of chemotherapy in the tumor cells, hence reducing the chances of drug resistance. Consequently, maximal cytoreductive surgery increases the effect of chemotherapy and eventually improves survival (1,5). The available data indicate a strong inverse correlation between survival and the residual tumor volume. Currently, a non-visible tumor is considered to be the optimal tumor volume for maximum survival (3). At the initial diagnosis of ovarian cancer, maximal surgical tumoral debulking and adjuvant chemotherapy are the standards of care. Surgical debulking in SC is not clearly defined due to a lack of prospective randomized trials and data that solely consist of retrospective studies. Berek *et al* reported the cases of 32 patients who underwent SC, and defined optimal debulking as a residual tumor of <1.5 cm (6). Optimally-debulked patients demonstrated a 20-month survival rate compared with a rate of 5 months for suboptimally-debulked patients. Chi *et al* presented 157 cases of patients with recurrent ovarian cancer who had undergone SC. The patients with residual tumors of <5 mm had a median survival of 56 months, whereas those with tumors of >5 mm survived for a median of 27 months (3). Optimal debulking by SC has been shown to be possible in two-thirds of patients (7). An increased disease-free interval prior to the secondary surgery and a small residual tumor load following the secondary surgery are highly-correlated with prolonged survival. The DESKTOP OVAR trial (the Arbeitsgemeinschaft Gynaekologische Onkologie Ovarian Committee, Descriptive Evaluation of pre-operative Selection KriTeria for OPerability in recurrent OVARian cancer), a multi-institutional retrospective study that proposed a selection criteria for females undergoing surgery for SC, attempted to classify and objectively select the appropriate patients (4). The residual tumor volume was the most significant prognostic factor that confirmed the results from the previous retrospective studies (8). The patients without macroscopic tumors showed longer survival rates than those with visible macroscopic tumors. The volume of the residual tumor was not significant. By incorporating the following three parameters of performance status, the presence of ascites and the outcome of the primary surgery/initial International Federation of Gynecology and Obstetrics (FIGO) stage, the recurrent ovarian cancer patients who had the optimal chance of complete surgical debulking with no residual tumor were objectively selected. Using the criteria established in the DESKTOP I trial, a prospective study, the DESKTOP II trial, confirmed the validity of these three parameters for selecting the female patients who underwent SC (9).

## 4. TC

Although recurrence following SC is often inevitable, there is no established treatment procedure. TC is an available option for the affected patients. Ideally, cytoreductive surgery should control the disease, diminish the complaints associated with the tumor load, increase survival and improve the quality of life without increasing morbidity. Surgeries for secondary recurrences are generally performed for palliation purposes to treat intestinal obstructions and pain, but since there is no requirement for cytoreduction, this is not classed as TC. Issues with regard to TC include selecting the appropriate candidates for the extensive surgery, determining the prognostic value and identifying the limits of how aggressive the surgery must be in order to achieve the best outcome.

## 5. Studies involving TC

The first study to evaluate TC was by Leitao *et al* (Memorial Sloan-Kettering Cancer Center, New York, NY, USA) (10), in which 26 patients with recurrent ovarian carcinoma were analyzed. Optimal debulking was defined as a residual tumor of <5 mm. Optimally-debulked patients had a median disease-specific survival (DSS) rate of 36 months, whereas this rate was 11 months for suboptimally-debulked patients. The survival rates of the optimally-debulked patients in this study was comparable to the results of other SC studies. The patients with longer disease-free intervals (>12 months) prior to TC survived for an average of 60 months, whereas the survival time of patients with shorter intervals was 15 months. In the multivariate analysis, the residual tumor following TC was the only independent factor associated with survival. Generally, in studies concerned with SC or TC, platinum resistance is an accepted exclusion criterion, although this may be a source of selection bias. In contrast, Leitao *et al* included 15 platinum-resistant patients (57% of the whole cohort) and 67% of these patients were successfully debulked. The median survival time following TC was 25 months. A second study with updated data was published in 2010 with a total of 77 patients, including the previously mentioned 26 patients and new patients with fallopian tube and peritoneal carcinoma (11). Nearly all the cases (92%) were optimally debulked to leave a residual tumor of <5 mm. Similar to the previous study, patients with platinum-resistant diseases (28%) were included. The median DSS, defined as the time between TC and mortality or last follow-up was 60 months for patients with optimal cytoreduction (non-visible tumor), 27 months for patients with a gross tumor of <5 mm and 13 months for patients with a residual tumor of >5 mm. The patients who had a recurrence interval of >24 months following SC were more likely to have optimal cytoreduction (no tumor left) than those with shorter recurrence-free intervals. The patients with platinum-sensitive diseases were more likely to have a complete tumor resection following TC than those with platinum-resistant diseases. Smaller tumors were more likely to be cytoreduced than larger (>5 cm) tumors. However, in the multivariate analysis, only the tumors with a single site of recurrence proved to be associated with a total resection. The extent of the debulking was the only significant prognostic factor for survival in the multivariate analysis. Adjuvant therapy following TC was not associated with an improved DSS. The post-operative complication rate was 26% and the majority of these complications were minor events. The study concluded that only a small group of patients with completely resectable tumors were considered appropriate candidates for TC (11).

The largest single institution study to evaluate TC was by Fotopoulou *et al* and involved 135 patients (12). Similar to the study by Leitao *et al*, patients with platinum-resistant diseases(20%) and those with ascites prior to TC (43%) were included. Patients with additional symptoms, including a chronic subileus, abscesses and pain, were also included. A tumor resection resulting in a non-visible status was achieved in 40% of the patients. Extensive procedures, including a small bowel resection (64%), large bowel resection (52%) and extensive peritonectomies (46%), were performed. The mortality rate within 30 days following the surgery was relatively high (5.8%). Unlike the findings in SC, the presence of peritoneal carcinomatosis in TC was not a risk factor associated with poor survival, regardless of the size of the post-operative residual tumor. Also, the previously established negative prognostic factors, including an advanced FIGO stage and the presence of ascites, did not decrease the survival rate. Therefore, the study recommended that patients who display these factors should not be excluded from TC surgery. The study also analyzed the distribution of the tumor in the upper, middle and lower abdominal cavity. In TC, the tumor usually involves at least two regions of the abdominal cavity, suggesting a diffuse involvement rather than a solitary recurrence site. The presence of a tumor in the middle abdomen and a diagnosis of peritoneal carcinomatosis in the multivariate analysis were negative factors associated with tumor resectability. Following TC, the overall survival rate for patients with a non-visible tumor was 37 months, whereas for those with a residual tumor of <1cm, the overall survival rate was 19 months. In the multivariate analysis, the overall survival rate was associated with a post-operative residual tumor of <1 cm. These parameters require challenging, highly-skilled surgical procedures in order to keep morbidity at a reasonable rate (12).

In a study involving cases from two institutions, Karam *et al* evaluated 47 patients who had undergone TC (13). Patients that were undergoing palliative procedures, including surgery for bowel obstructions, were excluded from the study. The surgery was performed on patients with longer disease-free intervals (e.g. 6 months) and those with a limited number of recurrences. Pre-operative computerized tomography scans revealed that the median number of disease sites was four. In approximately two-thirds of patients, optimal debulking with only a microscopic residual tumor was achieved, and 81% had a residual tumor of <1 cm. The presence of a macroscopic residual tumor was a poor prognostic factor. Patients with microscopic tumors following TC had an average 27-month survival rate, whereas patients with macroscopic tumors survived for 16 months. In contrast to the study by Fotopoulou *et al*, in the multivariate analysis, the presence of a diffuse disease was a negative predictor of survival, with optimal debulking having no effect. However, subsequent to the exclusion of patients with a diffuse disease, a subgroup analysis was performed in 34 patients. Optimal debulking was a significant factor in increasing the survival rate in the univariate and multivariate analyses (37 vs. 16 months). It was concluded that optimal TC extends the overall survival rate in patients with a limited range of diseases (13).

Gultekin *et al* evaluated the characteristics of 20 patients who had undergone TC (14). Patients with progressive diseases who were undergoing surgery for palliation were excluded. The tumor was resected to <2 cm in size in 12 patients, 7 of who had no visible disease remaining at the end of the TC. During a median follow-up period of 15 months, 13 patients were alive and three patients had not experienced any signs of recurrence. A total of three patients experienced perioperative complications with no surgery-related mortalities. The median survival was 32 months for patients with optimal TC compared with 6 months for patients with suboptimal TC. However, this difference was not statistically significant and may reflect the effect of the small sample size. There were no predictors for optimal debulking and no significant prognostic factors that affected the survival were detected upon analysis. The morbidity rate of 15% was lower than for other studies, since the surgery was less radical.

In a study from the Memorial Sloan Kettering Cancer Center, the value of quaternary cytoreduction (QC) was analyzed using 15 patients who had undergone surgery with the intent of surgical cytoreduction (15). A total of 14 patients had previously undergone optimal SC, which resulted in a residual tumor of <5 mm, and all patients had also previously undergone an optimal TC, resulting in 11 with no gross residual tumors. The median time between the third recurrence and the TC was 14 months, whereas between the TC and QC, the time interval was 24 months. Of the total number of patients, 20% were disease-free at the last follow-up, 25% had the disease but were alive and 50% had succumbed to the disease. A residual tumor of >1 cm and the number of recurrence sites (single vs. multiple) were associated with the survival time. The median DSS was 34 months for patients with a residual tumor of <1 cm and 10 months for patients with larger residual tumors. Platinum sensitivity did not affect the survival in QC, therefore it was not necessary to exclude platinum-resistant patients. QC was associated with certain complications, including one ileus resolved with conservative measures, three intra-abdominal abscesses, two of which required radiological drainage, and one colovesical fistula, which was managed with an ileal conduit and colostomy. There were no surgery-related mortalities. It was concluded that patients with resectable tumors that are ideally in one site may benefit from QC, provided that the tumor is debulked to a volume of ≤1 cm (15). Similarly, Fotopolou *et al* recently published a study with regard to QC in 49 patients, two-thirds of who exhibited peritoneal carcinomatosis. The survival time (43 vs. 13 months) increased significantly when no residual tumor was left. Patients who were administered post-operative chemotherapy had a significantly improved survival time compared with those who were not (40 vs. 12 months) (16).

**Figure 1:**
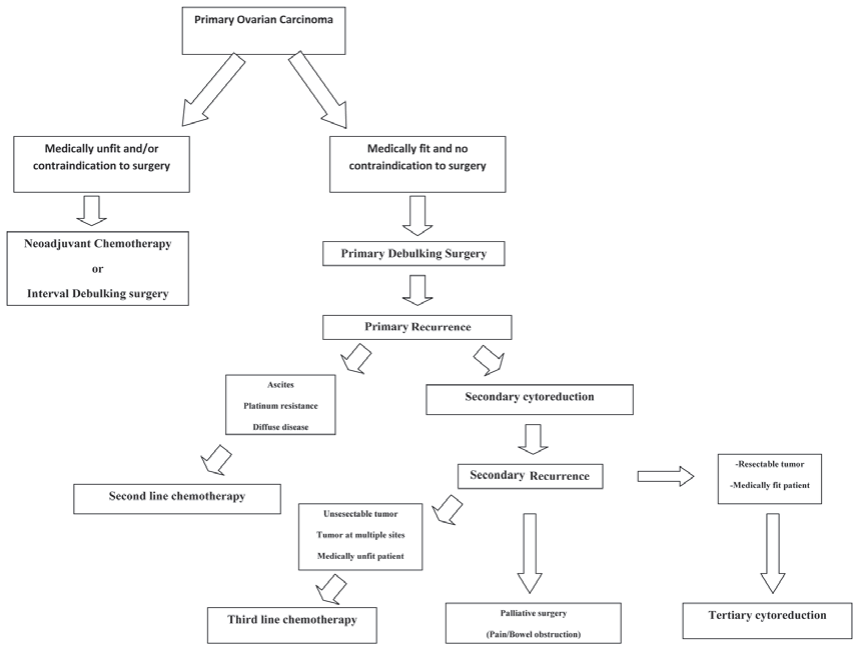
Algorithm showing the management options for primary ovarian cancer and primary and secondary recurrences.

In 2012, Hizli *et al* published a retrospective study of 23 patients who had undergone TC (17). A total of 12 patients with platinum-resistant diseases, diseases that were presumed unresectable by pre-operative imaging modalities or those with ascites were excluded. The median disease-free interval prior to SC was 26 months and the median interval between SC and TC was 21 months. More than one site of recurrence was identified in 82% of TC patients. Optimal debulking, defined as a residual tumor of <1 cm, was achieved in 65% of patients. No predictive factor for optimal debulking was identified and none of the variables were significant. The median follow-up period was 13 months, during which all but one patient remained alive. In the univariate analysis, only the outcome of TC (optimal vs. non-optimal) was associated with survival and there were no differences between the age of the patient or the time between progression. There were three perioperative morbidities, one of which was due to abdominal dehiscence and there were no surgery-related mortalities. Table I summarizes the data of the published TC studies and Fig. 1 shows the management options for primary ovarian cancer and primary and secondary recurrences.

Recently, a retrospective study involving 406 patients from 14 countries used data that were gathered from ovarian cancer patients who had undergone TC. A number of patients that were included in this study were also previously reported in other studies associated with TC. During a median follow-up period of 14 months, ~50% of the patients succumbed to the disease and another 49% experienced a new recurrence. The median overall survival rate was 26 months and the progression-free survival rate was 11 months. In concordance with other similar studies, the residual tumor status strongly correlated with the survival rate. Patients with no residual tumors had a longer overall survival time compared with patients with visible residual tumors. In contrast to other studies, a diagnosis of peritoneal carcinomatosis was not associated with a less favorable outcome. In the multivariate analysis, peritoneal carcinomatosis was associated with an incomplete tumor resection, but had no effect on overall survival (18).

Chemotherapy is generally administered to patients with secondary recurrences. In prospective trials, non-platinum agents resulted in complete responses in 3–4% of patients. For pegylated liposomal doxorubicin, the longest median survival time in patients with platinum-sensitive tumors was 27 months. For platinum-resistant patients, the response rates were worse, at less than one year (19,20). In the study by Leitao *et al*, platinum-resistant patients responded similarly to platinum-sensitive patients and the median survival was longer for platinum-resistant patients who underwent TC compared with those who were administered salvage chemotherapy (10). Therefore, platinum-resistant patients may also be candidates for TC, particularly if the tumor is resectable. In recent studies, Fotopoulou *et al* reported a survival advantage for patients who were administered third-line chemotherapy following TC over patients without chemotherapy (18,21).

Perioperative morbidity rates have been shown to range between 15 and 31% (11–14,17). In the study by Karam *et al*, the complication rate was 26% (13). A total of six patients experienced pulmonary embolisms and two presented with an enterocutaneous fistula. A further two patients were diagnosed with myocardial infarctions, and another two patients with rectovaginal and vesicovaginal fistulae, respectively. Despite these morbidities, there were no post-operative mortalities. In contrast, Fotopoulou *et al* reported a 5.8% mortality rate at 30 days post-surgery (12). The potential candidates for TC are expected to have high morbidity and mortality risks due to the fact that the majority of patients may have a diffuse disease, ascites or tumors extending to multiple sites, including the upper abdominal cavity. The patients should be informed of the high chances of operative morbidity and mortality and of the risk of a third operation. These procedures should be carried out in tertiary centers with multidisciplinary approaches, including highly-specialized centers with well-trained gynecological oncologists, gastroenterological surgeons and anesthesiologists.

There are certain common inherent drawbacks to all studies involving TC. Firstly, to date, there have been no randomized controlled trials that compared the various treatment modalities for recurrent ovarian cancer, since the current studies represent retrospective data. Secondly, the study groups consist of small numbers of patients with heterogenous characteristics. Finally, patient selection is not uniform and is based on the preferences of the surgeons rather than an objective criteria. Multicenter randomized studies with larger patient populations and more objective patient selection criteria are required to clarify these issues.

## 6. Conclusion

Conventional negative prognostic factors for SC, including ascites, an advanced FIGO stage and/or peritoneal carcinomatosis, do not apply to TC, but the post-operative residual tumor load is significant in predicting the outcome. Highly selective patients with completely resectable tumors may have a reasonable chance for maximal cytoreduction and an improved survival benefit. The aim should be to reduce the tumor so that no visible macroscopic residual volume is discernible and to select patients depending on this criteria. TC appears to have a favorable outcome and reasonable complication rates. Tertiary surgery is an available option for patients with recurrence in whom a complete tumor resection may be achieved.

## Figures and Tables

**Table I:** Retrospective studies with regard to TC.

First author (ref.)	Year	No. of patients	Percentage of platinum-sensitive patients	DFS^a^ (months)	DFS^b^ (months)	Major complication rate (%)	Operative mortality (%)	Complete tumor resection rate (%)	Independent factors associated with survival	Median tumor size (cm)	Multiple site recurrence rate (%)
Leitao *et al*(10)	2004	26	42	36	10	8	0	53	Optimal TC and TFI	5	57
Karam *et al*(13)	2007	47	0	24	16	14	0	64	Presence of diffuse peritoneal disease	5	NA
Gultekin *et al*(14)	2008	20	0	32	6	0	0	35	-	4	50
Shih *et al*(11)	2010	77	28	60	13	13	0	72	Extent of debulking	5	62
Fotopoulou *et al*(12)	2011	135	19	37	7	20	5.8	39	Complete tumor resection, interval to primary diagnosis >3 years and serous papillary histology	NA	85
Hizli *et al*(17)	2012	23	0	NA	NA	4	0	65	Complete tumor resection	4	83
Fotopoulou *et al*(18)	2013	406	38	49^c^	12^c^	26	3.2	54	High-grade histology, tumor residuals at 2nd and 3rd surgery, interval to second relapse, ascites, upper abdominal involvement, distant metastases and non-platinum third-line chemotherapy	NA	NA

a In optimally-debulked (no visible tumor) patients;

b in suboptimally-debulked patients;

c overall survival, instead of disease-free survival (DFS).

TC, tertiary cytoreduction; TFI, treatment-free interval; NA, not available.
